# Benzyl Alcohol/Salicylaldehyde-Type Polyketide Metabolites of Fungi: Sources, Biosynthesis, Biological Activities, and Synthesis

**DOI:** 10.3390/md21010019

**Published:** 2022-12-27

**Authors:** Mária Štiblariková, Angelika Lásiková, Tibor Gracza

**Affiliations:** Department of Organic Chemistry, Institute of Organic Chemistry, Catalysis and Petrochemistry, Slovak University of Technology, Radlinskeho 9, SK-812 37 Bratislava, Slovakia

**Keywords:** natural products, natural polyketides, secondary metabolites, biological activity, biosynthesis, total synthesis, salicylaldehydes, benzyl alcohols

## Abstract

Marine microorganisms are an important source of natural polyketides, which have become a significant reservoir of lead structures for drug design due to their diverse biological activities. In this review, we provide a summary of the resources, structures, biological activities, and proposed biosynthetic pathways of the benzyl alcohol/salicylaldehyde-type polyketides. In addition, the total syntheses of these secondary metabolites from their discoveries to the present day are presented. This review could be helpful for researchers in the total synthesis of complex natural products and the use of polyketide bioactive molecules for pharmacological purposes and applications in medicinal chemistry.

## 1. Introduction

Polyketides represent a vast group of secondary metabolites that are produced by certain living organisms to provide sustaining advantages for them. In terms of their chemical structures, Baerson and Rimando [1] described polyketide as a natural compound consisting of carbonyl and methylene groups alternating with each other. Polyketides and their derivatives represent an important group of compounds with remarkable diversity in both structure and function. Even though most polyketides are products of microbial origin (fungi and bacteria), these ubiquitous metabolites can be produced by a range of other organisms, such as plants, insects, lichens, mollusks, algae, sponges, crinoids, and dinoflagellates [1,2]. Polyketides are highly biologically active and multifaceted organic compounds. The structural diversity of polyketides allows them to have specific properties, such as pharmacological properties and biological activities. According to Dechert-Schmitt et al. [3], there is up to a five times higher probability of discovering a new polyketide-based drug compared to other groups of naturally occurring substances. Structurally smaller molecules of polyketide origin are among the top 20% of the best-selling drugs in the world [3]. This review provides a comprehensive summary of the resources, structures, biological activities, biosynthesis, and laboratory synthesis of benzyl alcohol/salicylaldehyde-type polyketide metabolites. The key criterion for the selection of the described compounds was the benzyl alcohol/salicylaldehyde structural pattern in the isolated secondary metabolites across the environment. As far as we can tell, known metabolites with the described structural characteristics are exclusively produced by fungi. A particularly significant source is fungi of marine origin. The metabolites obtained from terrestrial sources are included in order to cover all natural substances of interest produced by these species. This overview could be helpful for organic chemists in the search for new lead structures in drug design.

## 2. Benzyl Alcohol/Salicyladehyde-Type Secondary Cometabolites

### 2.1. Varitriol, Varioxirane, Andytriol, and Xanthones

#### 2.1.1. Isolation

The genus *Emericella*, first described by Berkeley [4], consists of thirty-four species [5,6], with their *Aspergillus* anamorphs assigned to the subgenus *Nidulantes* [7,8,9]. *Emericella* is likely to be present alongside with related *Aspergillus* anamorphs. The natural habitats of these fungi include soil, seeds, and vegetable matter. *Emericella* has ascomata embedded in masses of Hülle cells and red to violet ascospores, with a pronounced equator consisting of two, four, or multiple crests [10], but the ascospore ornamentation and particularly detailed morphology of the crests can vary.

**Figure 1:**
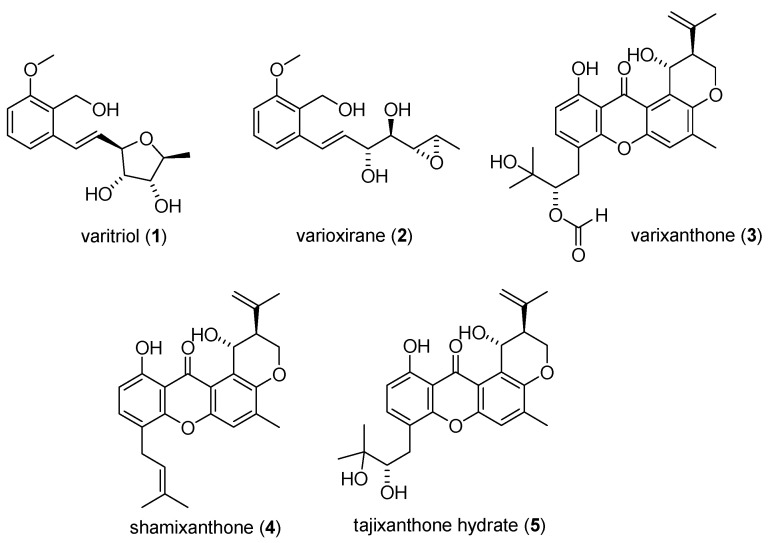
Structures of secondary metabolites **1**–**5** from *Emericella variecolor M75-2*.

In 2002, Malmstrøm and coworkers [11] described the isolation of four new metabolites from a marine-derived strain of the fungus *Emericella variecolor M75-2*, namely varitriol (**1**), varioxirane (**2**), and varixanthone (**3**) (Figure 1). Among the novel compounds, known metabolites, such as shamixanthone (**4**) and tajixanthone hydrate (**5**)**,** were also identified.

#### 2.1.2. Biosynthesis

The authors [11] suggested several plausible biosynthetic relationships between the isolated polyketide derivatives. A key intermediate appears to be 1-(2-hydroxymethyl)-3-methoxyphenyl)hepta-1,5-diene-3,4-diol (**6**), which was previously isolated from *Aspergillus variecolor* [12,13] (the imperfect state of *Emericella variecolor*). The metabolite was named andytriol after its first synthesis [14]. Andytriol (**6**) is structurally related to benzyl alcohol metabolites **1** and **2** of the *Emericella* genus [13].

**Scheme 1:**
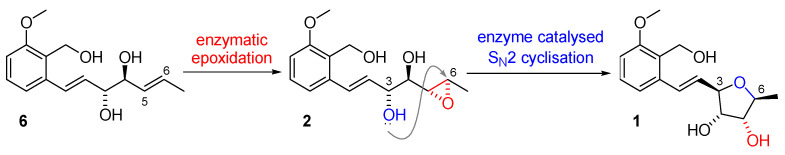
Biosynthesis of varitriol (**1**) by Malstrøm et al. [11].

Malmstrøm et al. [11] suggested that diene **6** could act as a precursor to varitriol (**1**) and that its biosynthesis probably proceeds via varioxirane (**2**). The enzymatic epoxidation of the C-5–C-6 double bond of **6** could provide epoxide **2**. Subsequent enzymatically catalyzed intramolecular S_N_2 oxirane ring opening at C-6 by the hydroxyl group in the C-3 position in varioxirane (**2**) could easily yield varitriol (**1**) (Scheme 1).

#### 2.1.3. Bioactivity

Metabolites isolated from the *Emericella variecolor M75-2* fungus were evaluated for antimicrobial activity against several Gram-positive and Gram-negative bacteria. Among the tested compounds, varixanthone (**3**) showed the best results. The MIC values for compound **3** were significantly lower than those for xanthone derivatives **4** and **5** [11]. 

Varitriol (**1**) and varixanthone (**3**) were additionally tested in vitro for their cytotoxic activities [11]. Varixanthone (**3**) was tested against mouse lymphoma (P388), human lung carcinoma (A549), and human colon carcinoma (HT29) cell lines. Unfortunately, compound **3** did not show any cytotoxic activity at the tested concentration. On the other hand, varitriol (**1**), which was tested in an NCI_60_ cell screen panel, demonstrated a more than 100-fold increased potency over the mean toxicity towards cell lines causing renal cancer (RXF 393, GI_50_ = 1.63 × 10^−7^ M), breast cancer (TD-47, GI_50_ = 2.10 × 10^−7^ M), and CNS cancer (SNB, GI_50_ = 2.44 × 10^−7^ M).

#### 2.1.4. Synthesis

The diversity of the biological activities of these relatively simple and structurally small secondary polyketide metabolites has attracted the interest of synthetic chemists. Among this group of natural compounds, varitriol (**1**) aroused the greatest interest, mainly for its biological properties. Therefore, most of the known syntheses are devoted to this metabolite.

##### Varitriol

**Scheme 2:**
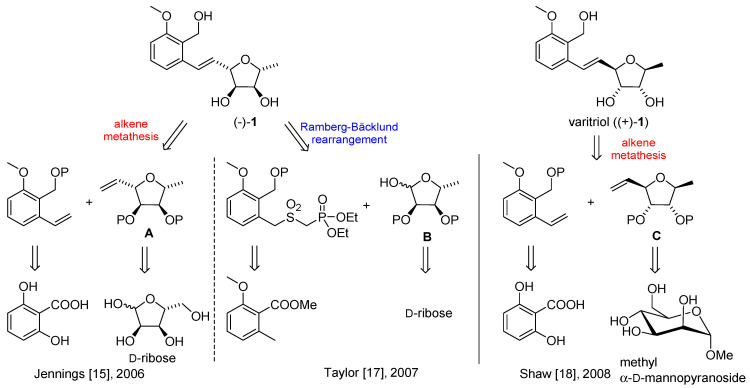
Retrosynthetic analyses of varitriol ((−)-**1**) and ((+)-**1**) [15,17,18].

Since the first synthesis [15] of the unnatural enantiomer (−)-**1**, accomplished by Jennings in 2006, about 22 total syntheses of varitriol have been reported in the literature. The review by Majik et al. [16] described different synthetic approaches towards **1** and its analogues in detail, covering the period up to 2013. The first two synthetic approaches commencing from d-ribose provided unnatural varitriol ((−)-**1**) (Scheme 2) [15]. Clemens and Jennings [15] achieved the total synthesis of (−)-**1** using cross-metathesis to link the carbohydrate **A** and aromatic moieties of the molecule. Taylor’s group reported a flexible approach to (−)-**1,** applying the HWE/conjugate addition/Ramberg-Bäcklund rearrangement sequence via intermediate **B [17]**. The aromatic part of (−)-**1** was obtained, either from 2,6-dihydroxybenzoic acid or methyl 2-methoxy-5-methylbenzoate. The first total synthesis of naturally occurring varitriol ((+)-**1**) utilizing methyl α-d-mannopyranoside was accomplished by Shaw and Kumar [18] (Scheme 2). The approach was based on an alkene metathesis of the corresponding styrene and tetrahydrofuran subunits. The key furanoside alkene **C** was obtained from methyl α-d-mannopyranoside.

**Scheme 3:**
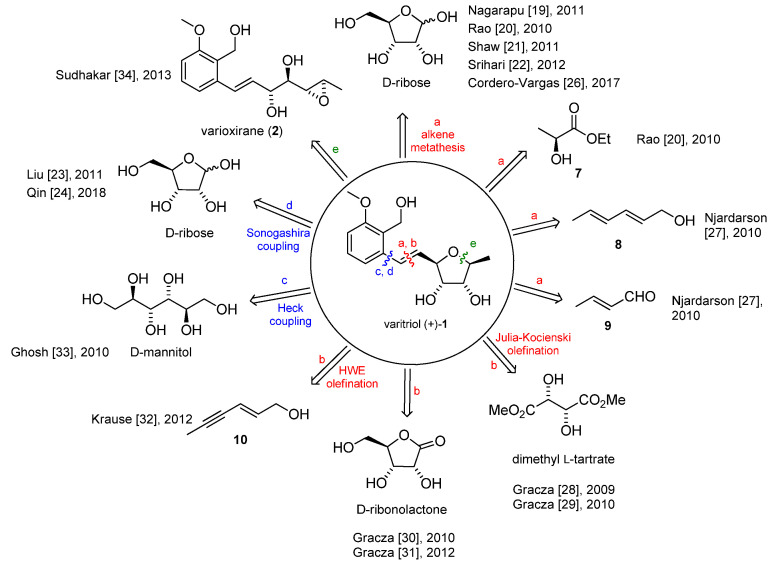
Synthetic strategies towards natural varitriol ((+)-**1**) [19,20,21,22,23,24,26,27,28,29,30,31,32,33,34].

After the definitive confirmation of the absolute configuration of natural varitriol (**1**), several other total syntheses were developed. These synthetic approaches usually involve multistep sequences and can be differentiated by the means of the disconnection of individual bonds in the varitriol (**1**) backbone (Scheme 3). The majority of the known strategies involve alkene metathesis for coupling the furanoside part and the corresponding styrene derivative (path a). The most common starting material for the synthesis of a furanoside-containing vinyl moiety (intermediate **A**/**B**) is d-ribose [19,20,21,22,23,24,25,26] Alternatively, an olefinic intermediate **C** can be obtained from ethyl (*S*)-lactate (**7**) [20] via a synthetic sequence involving epoxidation, cyclisation, and dihydroxylation reactions. Njardanson and coworkers [27] have described the syntheses of the carbohydrate part of (+)-**1** from 2,4-diene-heptanol (**8**) or but-2-enal (**9**) using the Cu-catalyzed vinyl oxirane ring expansion reaction. Another approach exploits the olefination of an aldehyde (path b). The Julia–Kocieński olefination was applied for the coupling of the furan-derivative-bearing sulfonyl group and the substituted benzaldehyde [28,29,30,31], which was easily available from 2,3-dimethylanisole. The required furanoside sulfone was prepared from dimethyl l-tartrate by employing the bicyclization strategy developed for the construction of 2,3-trans-substituted tetrahydrofurans [28,29]. The authors provided X-ray conformation of the absolute configuration of the natural (+)-varitriol (**1**) for the first time. The most efficient synthesis of natural varitriol (**1**) so far has been achieved from γ-d-ribonolactone and dimethylanisole (eight steps and 41% overall yield) [30]. The key steps of the route include a highly stereoselective introduction of the methyl group at C-1 and the installation of the side chain with the aromatic moiety using Julia–Kocieński olefination at C-5 of the starting carbon skeleton. This strategy was applied in the synthesis of novel varitriol analogues and a subsequent SAR study [31]. Krause et al. [32] reported the synthesis of (+)-**1** and its analogues. The authors utilized the Horner–Wittig–Emmons (HWE) condensation of the aromatic phosphonate and the chiral furanoside aldehyde, which was obtained from hept-2-en-4-ynol (**10**) using asymmetric synthesis. The linkage of the aromatic and sugar moieties was also achieved by a Heck C-C cross-coupling reaction between an aromatic triflate and an olefinic furanoside prepared from d-mannitol [33] (path c). Liu and coworkers [23] have developed a short synthesis of (+)-**1** by applying the Sonogashira reaction. The cross-coupling allowed the connection of the alkynic sugar moiety, obtained from d-ribose, and the aromatic triflate (path d). He and Qin [24] reported the formal synthesis of (+)-**1** using the Zn-catalyzed cross-coupling of the corresponding furanoside acetate with a benzene-derivative-bearing terminal alkynyl group (path d). Finally, varioxirane (**2**) was successfully converted into varitriol ((+)-**1**) via the intramolecular ring opening of the epoxide (path e) [34], supporting the proposed biosynthetic pathway [11]. 

##### Varioxirane and Andytriol

**Scheme 4:**
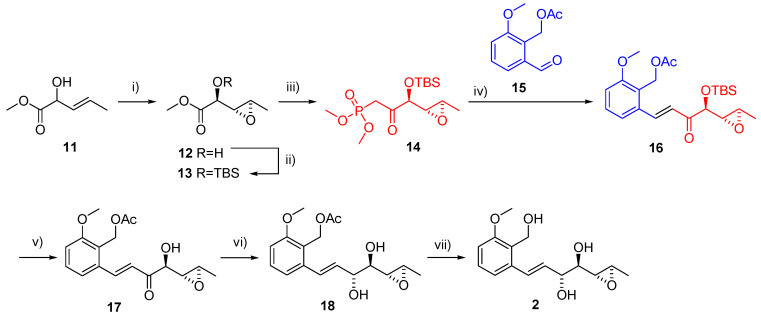
Synthesis of varioxirane (**2**). Reagents and conditions: (i) (−)-DIPT, TBHP, Ti(O*i*Pr)_4_, DCM, −20 °C, 8 h (42%); (ii) TBSOTf, 2,6-lutidine, DCM, 0 °C (96%); (iii) methyl phposphonate, *n*BuLi, THF, −78 °C (93%); (iv) KHDMS, THF, −78 °C → r.t., 24 h (74%); (v) HF.Py, THF, 0 °C → r.t. (81%); (vi) LiEt_3_BH, Et_2_O, −78 °C (82%); (vii) K_2_CO_3_, MeOH, 0 °C → r.t. (81%).

Sudhakar and Raghvaiah [34] developed the first synthesis of varioxirane (**2**) (Scheme 4). The synthesis commenced with the Sharpless kinetic resolution of α-hydroxy ester **11**, which afforded the epoxy alcohol **12** with the configuration corresponding to the target compound **2**. The key intermediate for Horner–Wittig–Emmons olefination, the phosphonate **14**, was obtained in two steps. A silylation of the secondary hydroxyl in **12** furnished **13**, which was treated with lithiated methyl phosphonate to afford **14**. The coupling of the phosphonate **14** with the aldehyde **15**, prepared from dimethylanisol [21], afforded ketone **16**. The desilylation of **16** with the subsequent reduction of ketone **17** using LiEt_3_BH in ether led to the desired anti-diol **18** with high diastereoselectivity (dr 32:1). The final deacetylation of **18** provided the target varioxirane (**2**) in seven reaction steps with a total yield of 15%.

**Scheme 5:**
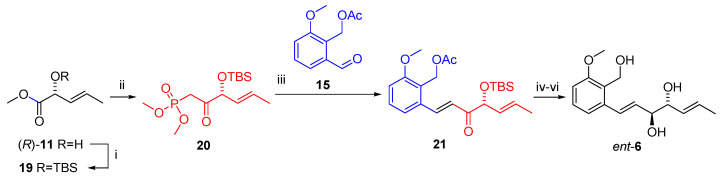
Synthesis of unnatural andytriol (*ent*-**6**). Reagents and conditions: (i) TBSOTf, 2,6-lutidine, DCM, 0 °C (98%); (ii) methylphosphonate, *n*BuLi, THF, −78 °C (97%); (iii) KHDMS, THF, −78 °C (82%); (iv) HF.Py, THF, 0 °C → r.t. (80%); (v) LiEt_3_BH, Et_2_O, −78 °C (dr, 32:1, 92%); (vi) K_2_CO_3_, MeOH, 0 °C → r.t. (85%).

The authors also carried out the synthesis of an enantiomer of the proposed biosynthetic precursor, (1*E*,3*S*,4*R*,*5E*)-1-(2-hydroxymethyl-3-methoxyphenyl)hepta-1,5-diene-3,4-diol (*ent*-**6**) (antipode of natural andytriol (**6**) [14]), from the unreacted α-hydroxy ester (*R*)**-11** isolated after the Sharpless kinetic resolution of the racemic **11** (Scheme 5). Thus, the silylation of (*R*)**-11** (er 98.6:1.4, HPLC) gave **19**, which was converted to the appropriate coupling partner **20** using methyl phosphonate with *n*BuLi in THF. Under HWE reaction conditions, **20** was coupled with aldehyde **15** to yield **21**. Lastly, a three-step sequence involving the removal of a TBS protecting group, the reduction of the carbonyl group using optimized reaction conditions (LiEt_3_BH, Et_2_O, −78 °C), and acetyl deprotection provided unnatural *ent*-**6**.

**Scheme 6:**
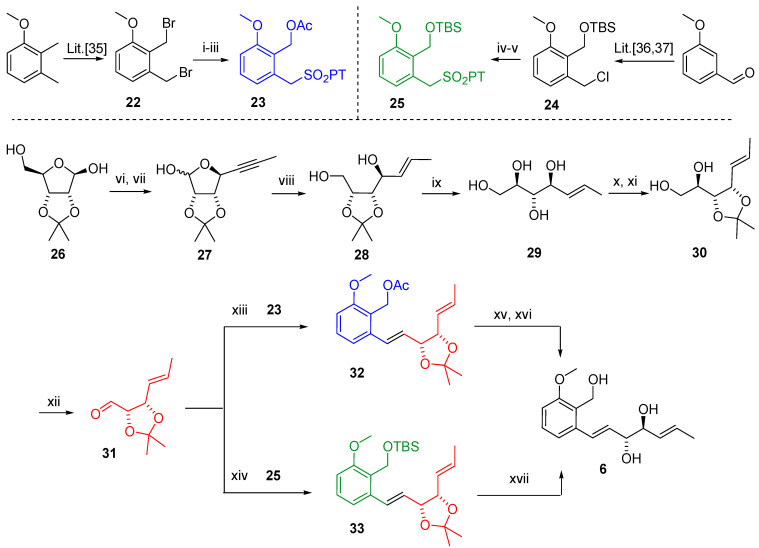
Synthesis of natural andytriol (**6**). Reagents and conditions: (i) AcONa, AcOH, 110 °C, 2 h (67%); (ii) KSPT, DMF, r.t., 15 h (85%); (iii) Mo(VI)/H_2_O_2_, MeOH, 0 °C to r.t., 10 h (82%); (iv) KSPT, DMF, r.t., 15 h (91%); (v) Mo(VI)/H_2_O_2_, MeOH, 0 °C to r.t., 15 h (77%); (vi) propyn-1-ylmagnesium bromide, THF, r.t., 1 h; (vii) NaIO_4_, *t*BuOH/H_2_O, r.t., 1.5 h (77% over 2 steps); (viii) LAH, THF, reflux, 2 h (88%); (ix) 60% AcOH, 60 °C, 2 h (96%); (x) DMP, CH_2_CI_2_, DOWEX marathon (H^+^), r.t., 2 h; (xi) 80% AcOH, 40 °C, 2 h (33%, 845 brs); (xii) NaIO_4_, *t*BuOH/H_2_O, r.t., 1.5 h; (xii) **30**, KHMDS, DME, −60 °C to r.t., 1.5 h (75%, *E*-isomer); (xiv) **32**, KHMDS, DME, −60 °C to r.t., 1.5 h (90%, *Z*/*E* 15:85); (xv) K_2_CO_3_, MeOH, r.t., 2 h; (xvi) 60% AcOH, 60 °C, 1.5 h (60% over 2 steps); (xvii) 60% AcOH, 60 °C, 4 h (73%) [35,36,37].

In 2014, Gracza and coworkers [14] described the first synthesis of natural andytriol (**6**) by employing the Julia–Kocieński olefination of aromatic sulfone **23**/**25** with corresponding hexenose **31** (Scheme 6). The requisite sulfones **23** and **25** were prepared by applying the known three-step protocol utilizing dibromide **22** [35] and chloride **24** [36,37] obtained from dimethyl anisole and 3-methoxy benzaldehyde, respectively. The aldehydic fragment for the olefination, hexenose **31**, was prepared from 2,3-*O*-isopropylidene-d-ribose (**26**) in seven steps (48% overall yield). The Grignard addition of propyn-1-ylmagnesium bromide at C-1 of **26,** followed by the oxidative cleavage of the vicinal diol using NaIO_4_, provided single diastereomer **27** in a 77% yield in a form of anomeric lactols. An LAH reduction of both the triple bond and carbonyl group in **27** afforded partially protected tetraol **28**. Its regioisomer, tetraol **30**, was prepared using a selective protection–deprotection sequence. Thus, the acidic hydrolysis of the 2,3-acetonide group in **28** afforded **29**. The introduction of 2,2-dimethoxypropane in the presence of Dowex H^+^ to all hydroxy groups, followed by the regioselective hydrolysis of the terminal acetonide, gave vicinal diol **30**. The mild oxidation of diol **30** using NaIO_4_, produced crude hexenal **31**, which was then subjected to Julia–Kocieński olefination with sulfones **23** and/or **25**. The final removal of all protecting groups furnished natural alcohol **6**. 

**Scheme 7:**
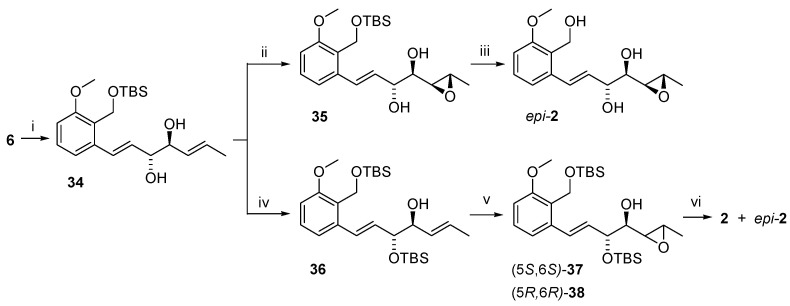
Biomimetic approach to varioxirane (**2**) and its 2,3-di-*epi*-varioxirane (*epi*-**2**). Reagents and conditions: (i) TBSCl, imidazole, CH_2_Cl_2_, r.t., 2 h (93%); (ii) d-DIPT, Ti(O*i*Pr)_4_, TBHP, CH_2_Cl_2_, −20 °C to 4 °C, 22 days (88%); (iii) TBAF, THF, r.t., 3 h (77%); (iv) TBSCl, imidazole, CH_2_Cl_2_, r.t., 7 days (68%); (v) d-DET, Ti(O*i*Pr)_4_, TBHP, CH_2_Cl_2_, −28 °C, 25 days (75%); (vi) TBAF, THF, r.t., 3 h (79%).

Furthermore, the authors explored a biomimetic epoxidation of **6** to construct the oxirane ring of the varioxirane (**2**) (Scheme 7). While the epoxidation of diene **6** using d-DET in a Katsuki–Sharpless reaction led to the unwanted 1,2-epoxy product, the silyl-protected diene **34** with d-DIPT, Ti(O*i*Pr)_4_, and TBHP in CH_2_Cl_2_ provided epoxide **35,** with high diastereoselectivity. A TBAF desilylation provided the undesired 2,3-di-*epi*-varioxirane (*epi*-**2**). The reaction of bis-silylated alcohol **36** using d-DET provided a 5:7 mixture of 2,3-epoxides **37** and **38** (75% yield). The deprotection of these epoxides using a TBAF reagent provided a mixture of **2** and *ent*-**2** in a ratio of 1:1 in a 79% yield.

### 2.2. Varioxiranols

#### 2.2.1. Isolation

**Figure 2:**
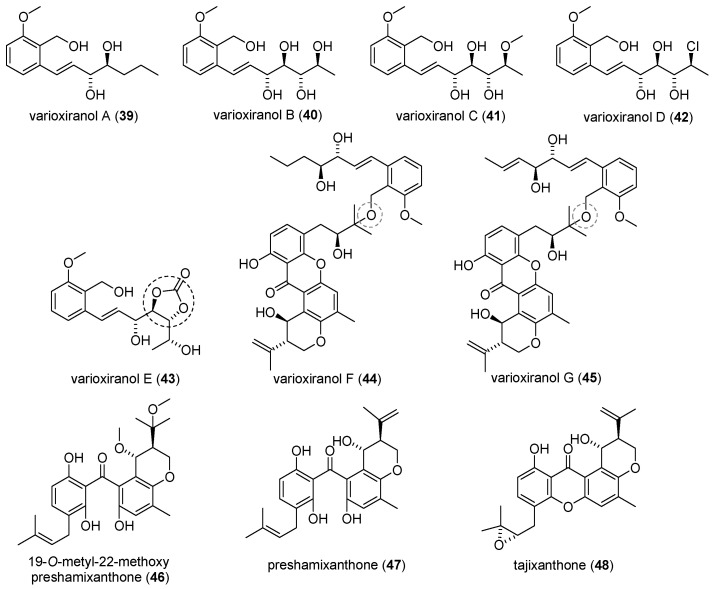
Structures of secondary metabolites **39**–**48** from *Emericella variecolor XSA-07-2*.

A chemical examination [38] of a sponge-derived microorganism, the fungus *Emericella variecolor XSA-07-2*, resulted in the isolation of eight new polyketide derivatives **39**–**46**, along with their previously characterized analogues **1**, **2**, **6**, **47**, and **48** (Figure 2). Structure elucidation revealed that metabolites **39**–**45** (varioxiranols A–G) comprise the same benzyl alcohol scaffold as andytriol (**6**), which can also be found in several other species of the fungus: *Emericella variecolor* (*Emericella variecolor XSA-07-02* [38], *Aspergillus variecolor*, and *Cinachyrella* sp. [12,13]). An examination of fungus *Emericella variecolor XSA-07-2* revealed other interesting findings, such as an unprecedented etheric bond between benzyl alcohol and a xanthone moiety in varioxiranols F (**44**) and G (**45**). The presence of a dioxolane group in derivative **43** is also worth mentioning, as it is generally quite rare and unusual in nature [39].

#### 2.2.2. Biosynthesis

**Scheme 8:**
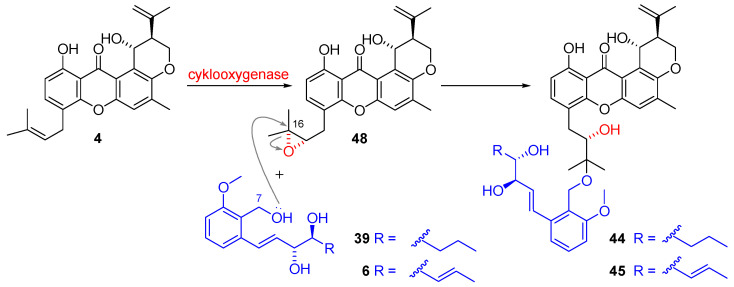
Formation of tajixanthone (**48**) and varioxiranols F (**44**) and G (**45**) [38,40].

The authors described varioxiranols F (**44**) and G (**45**) as products derived from the coupling of tajixanthone (**48**) with varioxiranol A (**39**) and andytriol (**6**), respectively (Scheme 8). The epoxide ring of tajixanthone (**48**) is formed by the oxidation of the carbon–carbon double bond at the prenyl group of **4**, which is mediated by cyclooxygenase [40]. The oxirane ring of **48** is then opened at the C-16 position by a nucleophilic attack of benzyl alcohol **6** or **39** to form varioxiranols F (**44**) and G (**45**). According to the authors, the enzymatic mediation of the epoxide ring opening is necessary to achieve the formation of ethers **44** and **45** with such high regioselectivity [38]. 

#### 2.2.3. Bioactivity

Isolated metabolites **1**, **2**, **6**, and **39**–**47** were tested for lipid-lowering effects against oleic acid (OA)-elicited lipid accumulation in HepG2 liver cells. Varioxiranol A (**39**), andytriol (**6**), and preshamixanthone (**47**) exhibited inhibitory effects at a dose of 10 μM, and they did not show any toxicity up to 100 μM towards the tested cells. Preshamixantone (**47**), as the metabolite with the highest activity levels, has shown an inhibitory effect comparable to the positive control simvastatin. In addition, a further evaluation revealed that compound **47** exhibited inhibition against intracellular triglyceride (TG) levels and dramatically reduced total cholesterol (TC) at a dose of 10 μM. Varioxiranol A (**39**) reduced TG levels with weak effects on TC, whereas andytriol (**6**) showed exactly opposite results, as it induced a reduction in TC, with a weak effect on TG levels. These surprising findings suggest there is a distinct mechanism for the anti-hyperlipidemic effects, since **6** differs from **39** only by having an extra double bond.

#### 2.2.4. Synthesis of Varioxiranol A and 4-Epi-Varioxiranol A

**Scheme 9:**
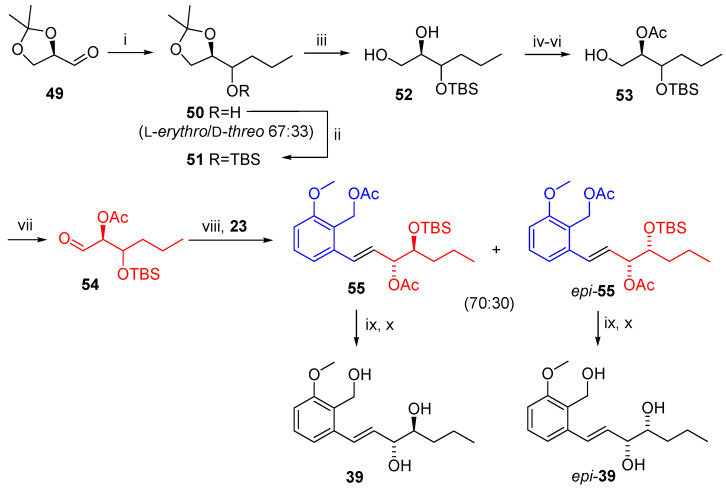
Synthesis of varioxiranol A (**39**) and 4-*epi*-varioxiranol A (*epi*-**39**). Reagents and conditions: (i) prop-1-ylmagnesium chloride, THF, Et_2_O, r.t., 1 h (71%); (ii) TBSCl, imidazole, CH_2_Cl_2_, 0 °C to r.t., 23 h (92%); (iii) TFA (50%), CH_2_Cl_2_, r.t., 1 h (93%); (iv) TrCl, Et_3_N, DMAP, CH_2_Cl_2_, 0 °C to r.t., 15 h (74%); (v) Ac_2_O, DMAP, CH_2_Cl_2_, r.t., 30 min (87%); (vi) HCOOH/Et_2_O (1/1), r.t., 50 min (66%); (vii) DMSO, (COCl)_2_, Et_3_N, CH_2_Cl_2_, −78 °C to r.t., 2.5 h; *(*viii) KHMDS, DME, sulfone **23**, CH_2_Cl_2_, −60 °C to r.t., 40 min (41% over 2 steps); (ix) K_2_CO_3_, MeOH, r.t., 2.5 h; (x) TBAF × 3H_2_O, THF, 0 °C to r.t., 4.5 h (92% of **39** and 57% of *epi*-**39** over 2 steps).

Lásiková and coworkers [41] have developed the only total synthesis of varioxiranol A (**39**) to date (Scheme 9). This chiral pool approach employed Julia–Kocieński olefination to connect the aromatic sulfone and the carbohydrate fragment. The synthesis of alkyl fragment **54** commenced from 2,3-*O*-isopropylidene d-glyceraldehyde (**49**), employing the Grignard addition, TBS protection, and acetonide deprotection to give a diastereomeric mixture of partially protected l-*erythro*/d-*threo* triols **52** in the ratio of 67:33. The aldehyde **54** was prepared using a selective protection–deprotection sequence involving a selective tritylation of the primary alcohol in **52**, followed by the acetylation of the secondary hydroxyl group and smooth tritylether hydrolysis using formic acid in ether furnishing alcohol **53**. The Swern oxidation of alcohol provided the desired aldehyde **54**, which was subjected to Julia–Kocieński coupling with sulfone **23**. The resulting *E*-alkenes **55**/*epi*-**55** were separated by MPLC and the final removal of all protecting groups, which were run in parallel with the pure diastereomers **55** and *epi*-**55**. The first basic hydrolysis of acetyl groups was followed by the treatment of the mixture with TBAF in THF, and finally flash chromatography purification furnished the target compounds **39** and *epi*-**39** in 92% and 57% yields, respectively. The absolute stereochemistry of both natural varioxiranol A (**39**) and its 4-epimer *epi*-**39** was confirmed by a single-crystal X-ray analysis.

### 2.3. Varioxiranediol

#### 2.3.1. Isolation

**Figure 3:**
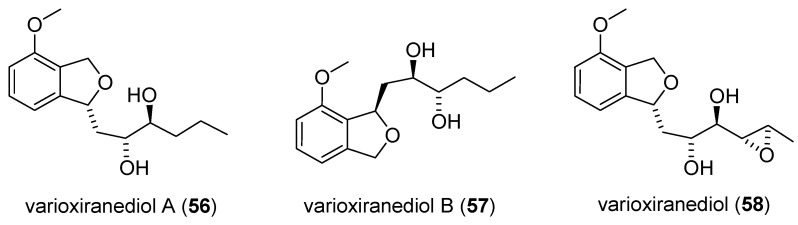
Structures of secondary metabolites **56**–**58** from *Emericella* sp. *TJ29*.

New strain of polyketide derivatives with benzyl alcohol scaffolds **56**, **57,** and **58,** along with previously established analogue **2** [11,42] was obtained by the bioassay-guided isolation of metabolites from cultures of the plant-derived fungus *Emericella* sp. *TJ29* (Figure 3) [42,43]. During the structural evaluation of **56**–**58,** a benzofuran motif appeared to be reoccurring, and the relative configuration of the stereogenic centers was further established based on spectroscopic experiments (HMBC, COSY, TOCSY, and NOESY) and an X-ray analysis [17,18]. The stereochemistry of the alkyl chain of polyketides **56**–**58** corresponds to the configuration of andytriol (**6**).

#### 2.3.2. Bioactivity

Wang, Xue, Zhang, and coworkers [43] performed an extensive biological screening against five antibiotic-resistant bacteria to try to identify promising novel antibiotics. Among the isolated compounds, varioxiranediol A (**56**) demonstrated the highest antibacterial activity against ESBL-producing *E. coli* and *P. aeruginosa*, with MICs of 4 μg/mL for both strains.

### 2.4. Pyricuol and Pyriculols

#### 2.4.1. Isolation

**Figure 4:**
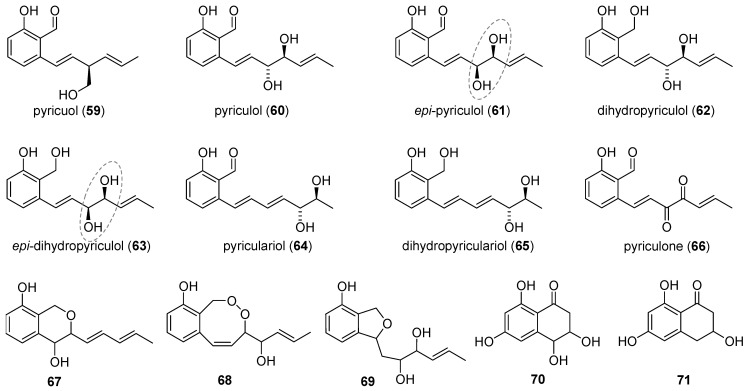
Structures of secondary metabolites **59**–**71** from *Magnaporthe grisea*.

In 1969, the fungus *Magnaphorthe grisea* (syn. *Pyricularia oryzae*) caused massive damage to a rice plant in Japan and other Asian countries. This fungus produces several different phytotoxic compounds **59**–**66** [40,44,45,46,47] that are structurally very similar to andytriol (**6**) (Figure 4). Among the secondary metabolites **59**–**71,** *epi*-dihydropyriculol (**63**) and *epi*-pyriculol (**61**) were isolated. Benzyl alcohols **63** and **61** represent the first case of secondary metabolites isolated from the studied genera of fungi in which a *syn*-arrangement of aliphatic hydroxyl groups was observed. 

#### 2.4.2. Biosynthesis

**Scheme 10:**
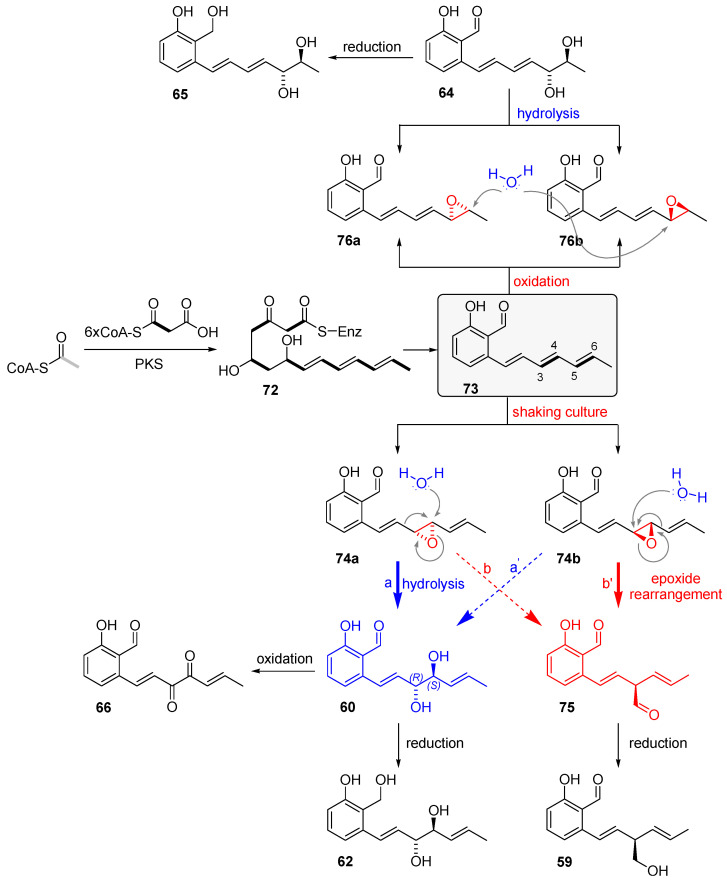
The biosynthetic pathway for salicylaldehyde-type phytotoxins by Tanaka, Kiyota et al. [48].

In 2011, Tanaka et al. [48] synthetized heptatrienylsalicylaldehyde (**73**) in a deuterium-labeled form as a plausible intermediate for the biosynthesis of metabolites **59**–**66**. Based on a biosynthetic study, the authors proposed a biosynthetic pathway of polyketides derivatives **59**–**66** (Scheme 10). Their findings showed that the biosynthesis of **73** involves the condensation of acetyl-CoA with malonyl-CoA and the subsequent aromatization of polyketide **72**. The resulting triene **73** appears to be biosynthetic precursor for salicylaldehyde-type metabolites [49]. An epoxidation of the C-5–C-6 double bond of **73,** followed by the hydrolysis of the oxirane ring in **76a** and **76b,** gives pyriculariol (**64**). A reduction of the aromatic aldehyde **64** affords dihydropyriculariol (**65**). Similarly, a fermentation by a shaking culture allows the epoxidation of the 3,4 double bond of **73**, which leads to the formation of epoxides **74a** and/or **74b**. Epoxy rearrangement in **74a** and/or **74b** (routes b and b′), followed by the regiospecific reduction of the aliphatic aldehyde **75**, provides pyricuol (**59**). The hydrolysis of epoxides **74a** and **74b** provides pyriculol (**60**) with the 3*R*,4*S* absolute configuration (routes a and a′). The authors claimed that the biosynthesis of **60** could be performed by asymmetric epoxidation followed by regioselective hydrolysis and/or non-stereoselective oxidation followed by stereospecific hydration. The oxidation and reduction of **60** provide pyriculone (**66**) and dihydropyriculol (**62**), respectively. Interestingly, the only structural difference between dihydropyriculol (**62**) and the secondary metabolite of *Emericella* species, andytriol (**6**), is the presence of a methyl group attached to the phenolic OH.

Recently, the gene encoding a polyketide synthase, *MoPKS19*, in *M. oryzea* was identified to be responsible for the biosynthesis of pyriculol and related compounds [47] *MoPKS19* resides in a gene cluster that contains additional biosynthetic enzymes found in fungal PKS pathways, including oxidoreductases and cupin-domain-containing proteins. The roles of most of the other enzymes in this cluster are unknown.

#### 2.4.3. Bioactivity

*Magnaporthe* species are a huge threat for rice plants. Their metabolites have strong phytotoxic properties and cause the inhibition of growth and leaf damage at levels as low as 60–500 ppm [50]. An investigation of the metabolites showed that pyriculol (**60**) is responsible for the disease. The results suggested that salicylaldehyde functionality brings about the phytotoxicity since benzyl alcohols **62** and **63** were described as non-phytotoxic [51]. 

#### 2.4.4. Synthesis

##### Pyriculol

**Scheme 11:**
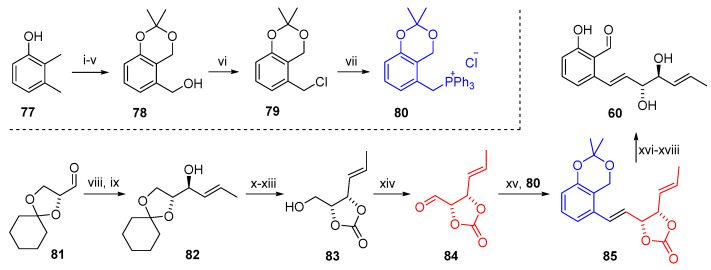
Synthesis of pyriculol (**60**). Reagents and conditions: (i) Ac_2_O, pyridine; (ii) NBS, benzoyl peroxide, CCl_4_, reflux, 1 h (92%); (iii) AcOH, AcOK, reflux, 3 h (85%); (iv) LAH, THF, 0 °C → reflux, 6 h (83%); (v) *p*-TsOH, acetone, r.t., overnight (73%); (vi) PPh_3_, CCl_4_, reflux, 10 h (73%); (vii) PPh_3_, xylene, reflux, 15 h (67%); (viii) propyne, BuLi, ZnCl_2_, THF, −70 → −10 °C, overnight (81%); (ix) LiAlH_4_, THF, r.t., 2 h (92%); (x) 70% AcOH; (xi) Ph_3_CCl, pyridine (68%); (xii) (MeO)_2_CO, NaH, −10 °C, 4 h (81%); (xiii) *p*-TsOH, MeOH-THF-H_2_O (91%); (xiv) (COCl)_2_, DMSO, Et_3_N, DCM; (xv) BuLi, Et_2_O, −10 °C → r. t., 5 h (32% over 2 steps); (xvi) p-TsOH, aq. THF, 60 °C, 4 h (82%); (xvii) MnO_2_, DCM, r.t., 30 min (72%); (xviii) K_2_CO_3_, MeOH-H_2_O, r.t., 4 h (57%).

Among the phytotoxic metabolites [39,44,45,46,47] produced by *Pyricularia oryzae*, three were synthesized, and their definitive structures as well as the absolute configurations were determined [52,53,54,55,56,57,58]. First, pyriculol (**60**) [39], a compound bearing a salicylaldehyde moiety with an unsaturated side chain containing two stereogenic centers, was prepared [52,53]. The authors developed a convergent approach based on the preparation of aromatic and aliphatic fragments and their subsequent condensation (Scheme 11). The aromatic synthon was prepared from 2,3-dimethylphenol (**77**) in accordance with the previously reported protocol [59]. The phenol **77** was acetylated and successively treated with *N*-bromosuccinimide, sodium acetate, potassium hydroxide or lithium aluminum hydride, and 2,2-dimethoxypropane or acetone to give **78**. The resulting hydroxyacetonide **78** was converted to a corresponding chloride **79** using triphenylphosphine-carbon tetrachlochloride, which was transformed into phosphonium salt **80**. The synthesis of the side chain moiety commenced with 2,3-*O*-cyclohexylidene-d-glyceraldehyde (**81**), which afforded upon prop-1-ynyllithium addition, followed by an LAH reduction of the carbon–carbon triple bond, a separable diastereomeric mixture of *E*-allylic alcohols **82** (*erythro*/*threo*, 2.1:1). In addition, the *threo*-diastereomer was transformed into *erythro*-triol using a Mitsunobu inversion. The *erythro* product **82** was converted to the carbonate **84** through the following sequence: the removal of a cyclohexylidene group from **82** with 70% acetic acid, the tritylation of a primary hydroxyl group, the cyclic carbonation of a residual 2,3-diol with dimethyl carbonate and sodium hydride, and finally the acidic hydrolysis of the trityl protecting group, providing the alcohol **83**. The Swern oxidation of **83** gave aldehyde **84**, which was used without further purification in Wittig olefination with **80**. A mixture of geometrical isomers **85** (*E*/*Z*, 4:1) was obtained in a 32% yield over two steps. The desired *E*-isomer was obtained by thin-layer chromatography. The acetonide protection on the aromatic ring was removed with *p*-TsOH upon refluxing in an aqueous THF solution to give a hydroxymethyl moiety. Finally, the oxidation of a primary alcohol with activated manganese dioxide, followed by the hydrolysis of a carbonate protecting group, gave pyriculol (**60**).

##### Pyricuol

**Scheme 12:**
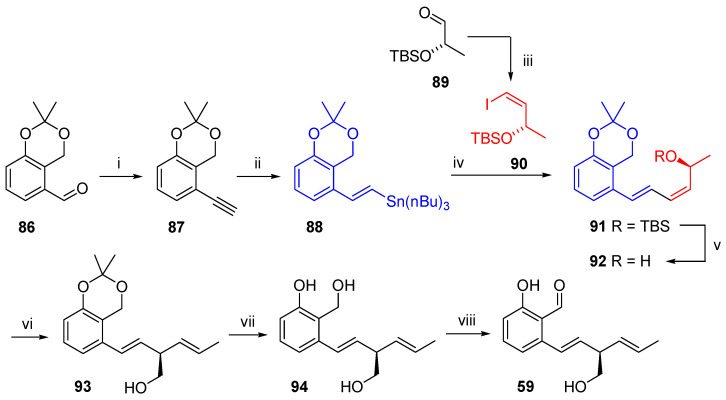
Synthesis of pyricuol (**59**). Reagents and conditions: (i) dimethyl-1-diazo-2-oxopropylphoosphonate, K_2_CO_3_, MeOH (98%); (ii) Bu_3_SnH, CuCN, BuLi, THF, −76 °C → −37 °C; (iii) (Ph_3_P^+^CH_2_I)I^−^, NaHMDS, HMPA, THF, −78 °C (41%); (iv) Pd_2_dba_3_, AsPh_3_, CuI, DMF, r.t.; (v) TBAF, THF, r.t., 2 h (20% over 3 steps); (vi) KH, Bu_3_SnCH_2_I, THF, r.t., then *n*BuLi, THF, −79 °C (69%); (vii) *p*-TsOH, MeOH–HCl (94%); (viii) MnO_2_, DMSO, 60 °C, 1 h (75%).

In 2003, Kiyota and coworkers [54] developed a synthesis of pyricuol (**59**), which allowed the confirmation of its structure. However, the absolute configuration remained undetermined. Even though its following syntheses, commencing from (*R*)-lactate [55] and/or l-serine [56] provided the unnatural enantiomer (*S*)-**59**, they allowed the confirmation of the absolute stereochemistry of the natural pyricuol (**59**). Based on these results, the authors [57] successfully carried out the first synthesis of natural pyricuol (**59**) employing Stille coupling and [2,3]-Wittig rearrangement reactions as the key steps (Scheme 12). Aromatic fragment **88,** bearing a tributyltin group, was obtained from the known aldehyde **86** [60]. The treatment of **86** with Ohira–Bestmann [61,62] reagent, followed by hydrostannylation of **87** using Bu_3_SnH/CuCN/BuLi in THF, gave styryltin compound **88**. The partner for the C-C cross-coupling reaction, *Z*-vinyl iodide **90**, was prepared from the corresponding aldehyde **89** [63] via a Wittig olefination reaction with (Ph_3_P^+^CH_2_I)I^−^ in the presence of NaHMDS. An undesired *E*-isomer was removed using column chromatography. The Stille coupling of **88** with **90** afforded aromatic diene **91**. The TBAF removal of the silyl protecting group provided dienol **92** in a 20% yield over three steps. Next, the free hydroxyl group was etherified with Bu_3_SnCH_2_I, and the resulting stannylmethyl ether was treated with *n*BuLi, giving a [2,3]-Wittig-rearranged product **93**. The acidic hydrolysis of **93,** yielding **94**, followed by the selective oxidation of the benzylic hydroxy group with MnO_2_, furnished natural (*R*)-pyricuol (**59**).

##### Pyriculariol

**Scheme 13:**
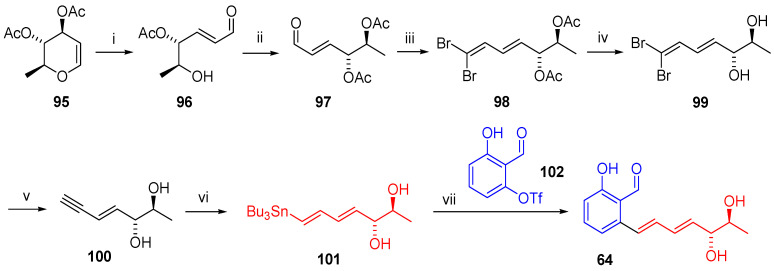
Synthesis of pyriculariol (**64**). Reagents and conditions: (i) [65], HgSO_4_, 0,05 M H_2_SO_4_/dioxane,/acetone (7:1:1.2), 25 °C, 3 h (93%); (ii) Ac_2_O, Py (85%, 2 steps); (iii) PPh_3_, CBr_4_, CH_2_Cl_2_ (54%); (iv) K_2_CO_3_, MeOH (99%); (v) BuLi, THF −78 °C (75%); (vi) Bu_3_SnH, AIBN, toluene reflux 33%); (vii) Pd_2_dba_3_, AsPh_3_, LiCl, DMF, microwave irradiation, −78 → 33 °C (69%).

In 2009, Kiyota and coworkers [58] reported the first total synthesis of (-)-pyriculariol (**64**). The synthesis of the alkenyl chain commenced with a Ferrier reaction of l-rhamnal diacetate (**95**) [64] providing aliphatic **96**, which upon the acetylation of the free hydroxyl group provided aldehyde **97** (Scheme 13). A Corey–Fuchs reaction using PPh_3_/CBr_4_, followed by the basic hydrolysis of both acetyl protecting groups in **98**, afforded diol **99**. The treatment of **99** with *n*BuLi gave enynediol **100**, which was converted into dienyl stannane **101** by radical-promoted hydrostannylation with Bu_3_SnH. Finally, two fragments (stannyl compound **101** and aromatic triflate **102** [64]) were coupled using the abovementioned procedure (Stille coupling) employing Pd_2_dba_3_, AsPh_3_, and LiCl in dimethylformamide under microwave irradiation, which allowed the formation of the desired product **64**.

### 2.5. Sordarials

#### 2.5.1. Isolation and Identification of Metabolites

**Figure 5:**
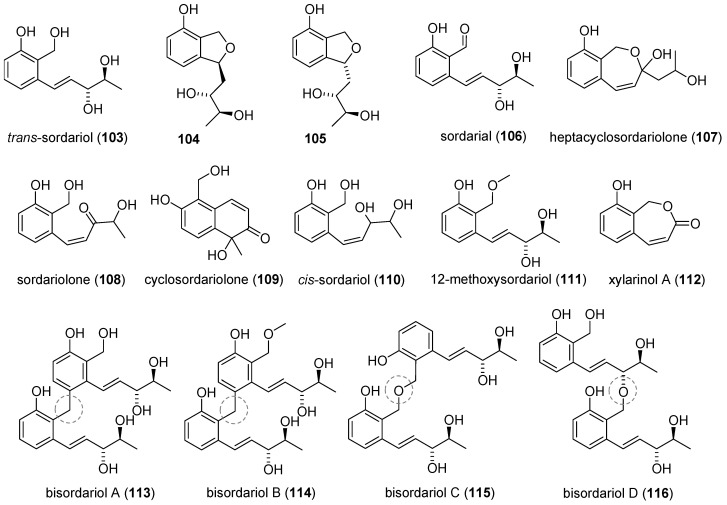
Structures of secondary metabolites **103**–**116** from *Sordaria macrospora*.

A group of polyketide metabolites was isolated from the endophytic fungus *Sordaria macrospora* (Figure 5) [50,51,65]. Among the previously characterized polyketides **6**, **60,** and **62**, new benzyl alcohol derivatives, trans-sordariol (**103**) and two isobenzofuranyl derivatives (**104** and **105),** were identified in the metabolite mixture obtained from the medium and the mycelium extracts [50]. An additional thorough reinvestigation of the *S. macrospora* broth extracts afforded sordarial (**106**), heptacyclosordariolone (**107**), sordariolone (**108**), cyclosordariolone (**109**), and cis-sordariol (**110**) [51]. 

Yang, Kong, and coworkers [65] described the structure elucidation and antioxidant activities of polyketides **109**–**116** isolated from solid cultures of *S. macrospora* that were obtained from the bark of *Ilex comuta*. The hexaketides from *S. macrospora* are chemically related to the heptaketide pyriculol family, in which all products, except for **109**, contain a *1*,*2*,*3-*trisubstituted benzene ring. Another typical structural motif, an alkenyl vicinal diol attached to an aromatic ring, was observed in these metabolites. The absolute configuration 3*R*,4*S* was established for all determined structures. Moreover, the dimeric metabolites **48**–**51** were identified for the first time. Bisordariol A (**113**) and B (**114**) can be described as sordariol dimers connected via a new C-C bond, while bisordarial C (**115**) and D (**116**) represent novel ether-bond-linked sordariol dimers.

#### 2.5.2. Biosynthesis

**Scheme 14:**
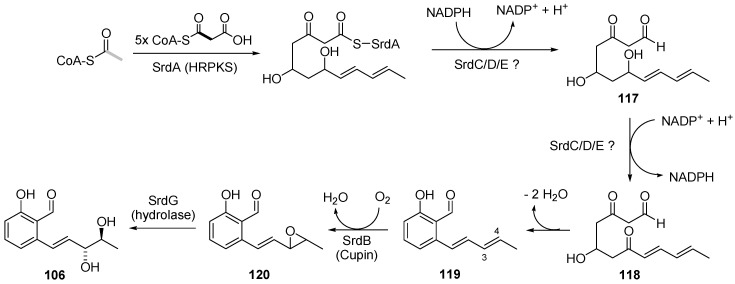
Proposed biosynthetic pathway of sordarial (**106**) [47].

Scheme 14 shows the proposed biosynthesis [47] of sordarial (**106**). The experimental results could indicate that the *srd* gene cluster (*srdA*, *srdB*, *srdC*, *srdD*, *srdE*, and *srdG*) is responsible for the synthesis of sordarial. The authors suggested that a polyketide chain **117** is formed by the condensation of one molecule of acetyl-CoA and five molecules of malonyl-CoA. Formed hexaketide is then reductively released from PKS as an aldehyde **117**, and a corresponding hydroxyl group is enzymatically oxidized to give **118**. Ketone formation enables the intramolecular aldol condensation, which is followed by dehydration to yield salicylaldehyde **119**. The selective epoxidation of the 3,4 double bond in **119** and the hydrolysis of the oxirane ring in **120** affords sordarial (**106**). 

Although the biosynthesis of **103** has not been described so far, the formation of sordariol (**103**) by the reduction of the aldehyde group of **106** is conceivable.

#### 2.5.3. Bioactivity

Metabolites of *S. macrospora* were tested for their antioxidant activities using ABTS radical scavenging assays with Trolox as a positive control [65,66]. Sordariol (**103**) was not phytotoxic towards race roots or race shoots [50] and did not show any immunosuppressive activity [66]. In contrast to the monomeric compounds of this origin, the dimers **113**–**115** exhibited more potent effects. The authors suggested that the way in which the dimers are connected affects the ABTS radical scavenging abilities, considering that bisordariol D (**51**) showed a significantly higher value of EC_50_ (57.6 ± 7.5 mM) compared to the rest of the dimers **113**–**115**. 

Additionally, the compounds were tested for their cytotoxicity against human tumor cell lines U2OS, MCF-7, and HepG2. No activity was observed at concentrations up to 50.0 mM [31].

### 2.6. Agropyrenols, Agropyrenals, and Agropyrenone

#### 2.6.1. Isolation and Identification

**Figure 6:**
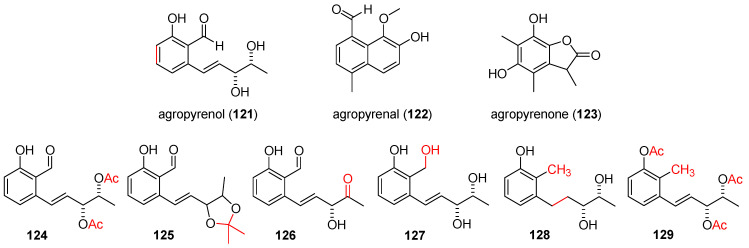
Structures of natural metabolites **121**–**123** and synthetic derivatives **124**–**129**.

A phytotoxicity-guided isolation of metabolites from the strain *Ascochyta agropyrina var. nana*, a fungal pathogen of the perennial weed *Elytriga repens*, afforded several toxins in the liquid medium (Figure 6) [67]. The main metabolite, agropyrenol (**121**), was determined to be a *syn*-diastereomer of sordarial (**106**). Two other minor metabolites (**122** and **123)** were isolated from the less polar fraction during the column chromatography separation. They were characterized as trisubstituted naphthalene carbaldehyde **122** and pentasubstituted 3*H*-benzofuranone **123**, respectively.

#### 2.6.2. Bioactivity

Preliminary tests on leaves of several weed species have disclosed the relatively low phytotoxicity of agropyrenol (**121**) and agropyrenal (**122**), while agropyrenone (**123**) was described as inactive. None of the metabolites showed antibiotic, fungicidal, or zootoxic activity [67]. Considering the simple chemical structure of agropyrenol (**121**) and the identified biological properties, the authors decided to perform an SAR study of this metabolite to determine its potential as a new natural herbicide [68]. Compounds **124**–**129**, prepared by the modification of the functional groups of **121** (Figure 6), were assayed for phytotoxic, antibacterial, and zootoxic activities, and the SAR was examined. The results of the tests on nonhost weedy and agrarian plants, fungi, Gram-positive and Gram-negative bacteria, and brine shrimp larvae are summarized in Table 1. The data show that the presence of the double bond and the diol in the aliphatic chain as well as the aldehyde group attached to the aromatic ring seems to play a key role in the biological activity of agropyrenol (**121**). The introduction of protecting groups (compounds **124**, **125**, and **129**) did not cause any significant loss of the biological activity, as the authors assumed that their hydrolysis occurs at a physiological pH. These less polar derivatives of agropyrenol (**121**) also showed significant zootoxic and slight antimicrobial activities. The results from this biological activity evaluation could be useful in the testing of other secondary metabolites.

### 2.7. Heterocornols

#### 2.7.1. Isolation and Identification

**Figure 7:**
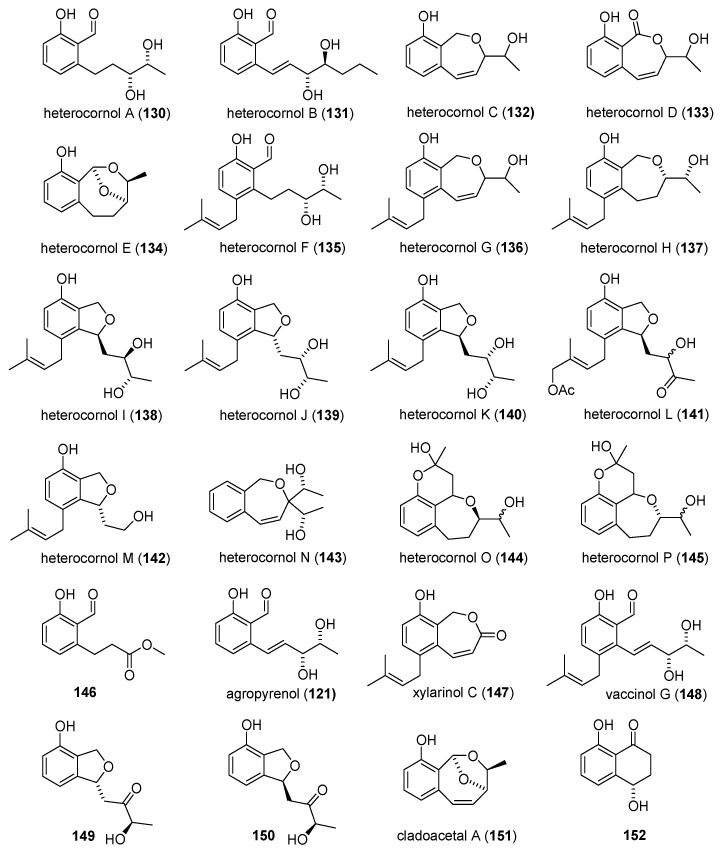
Structures of secondary metabolites **130**–**152** from *Pestalotiopsis heterocornis*.

Genus *Pestalotiopsis* represents another producent of salicylaldehyde-type metabolites. In 2017, Huang, Han, and coworkers [68] described twelve new polyketides derived from salicylaldehyde. The investigation of the fermentation extract of *Pestalotiopsis heterocornis* led to the isolation of heterocornols A–E (**130**–**134**) and heterocornols F–L (**135**–**141**), together with the known compounds methyl-(2-formyl-3-hydroxyphenyl)propanoate (**146**), cladoacetal A (**151**) [69], xylarinol C (**147**) [70], agropyrenol (**121**) [67], vaccinol G (**148**) [71], (*R*)-3-hydroxy-1-[(*R*)-4-hydroxy-1,3-dihydroisobenzofuran-1-yl]butan-2-one (**149**) [72], and (*R*)-3-hydroxy-1-[(*S*)-4-hydroxy-1,3-dihydroisobenzofuran-1-yl]butan-2-one (**150**) [72] (Figure 7). Another four new polyketides of this type, heterocornols M (**142**) and N (**143**) and a pair of epimers, heterocornols O (**144**) and P (**145**), were isolated from a refermented strain of *P. heterocornis* in the same culture medium [73] (Figure 7).

#### 2.7.2. Biosynthesis

**Scheme 15:**
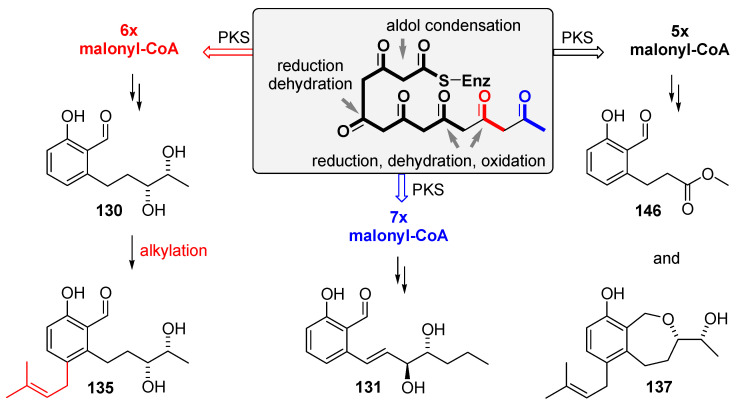
Proposed biosynthetic pathway of heterocornols [69].

Polyketides are common biosynthetic precursors in the synthesis of aromatics and macrolides in microorganisms. Based on the structural similarities of the heterocornols to sordarial (**106**), the biosynthesis of which is described (Scheme 14) [47] it can be assumed that the biosynthesis of these secondary metabolites proceeds very similarly. The authors [69] suggest that the identified secondary metabolites from *Pestalotiopsis heterocornis* were formed from complementary polyketide precursors in the presence of PKSs. Scheme 15 shows the reaction centers where the enzymatic transformations occur. The length of the chain and the structures of the final polyketide derivatives depend on the number of malonyl-CoAs that participate the condensation reaction. 

#### 2.7.3. Bioactivity

Metabolites of *P. heterocornis* were evaluated by an MTT assay for their cytotoxic activities against four human cancer cell lines, including a human gastric carcinoma (BGC-823), a human large-cell lung carcinoma (H460), a human prostate cancer (PC-3), and a human hepatocellular carcinoma (SMMC-7721) [68]. 6-Alkylsalicylaldehydes **121, 130, 131, 135, 146,** and **148** and benzooxepines **132, 136,** and **137** showed moderate cytotoxicity, with IC_50_ values of 15–100 μM with adriamycin assayed as a positive control. In the antimicrobial assay, the MIC values for the same compounds were evaluated between 25 and 100 μg/mL for the pathogens *Staphylococcus aureus* and *Bacillus subtilis*. The antifungal properties of the isolated derivatives appeared to be dependent on the presence of a pent-4-ene-2,3-diol block. The derivatives **132, 121, 136,** and **148** showed moderate antifungal activity against *Candida parapsilosis* and *Cryptococcus neofromans* at 100 μg/mL (Table 2).

Heterocornols M–P (**142**–**145**), together with polyketide derivatives **149**, **150,** and **152**, were evaluated for their cytotoxicity against a carcinoma cell line (Ichikawa), gastric cancer (BGC-823), liver cancer (HepG2), and kidney cancer (7860). The tested metabolites showed activity, with IC_50_ values of 20.4–94.2 μM. Compounds **143** and **152** showed no cytotoxic activity [73].

## 3. Conclusions

To date, more than 100 polyketides containing either benzyl alcohol or a salicylaldehyde aromatic moiety have been isolated and characterized. These secondary metabolites are mainly produced by the fungus *Emericella variecolor*, which is widely distributed in marine and terrestrial environments. Structurally related polyketides derivatives of the benzyl alcohol/salicylaldehyde-type were obtained from other genera of the ascomycetes, namely *Sordaria*, *Magnaporthe*, *Pestalotipsis*, and *Ascochyta*. The biosyntheses of these polyketide structures involve the condensation of acetyl-CoA with malonyl-CoA in the presence of the specific polyketide synthases (PKS) and the subsequent aromatization of the polyketide chain. The resulting salicylaldehyde derivative with an aliphatic polyenyl substituent appears to be key for the biosynthesis of salicylaldehyde- and benzyl alcohol-type metabolites. The reported polyketide derivatives exhibited valuable bioactivities, particularly those of marine origin. The highest cytotoxicity towards the human cell line panel was demonstrated with varitriol (**1**), while agropyrenol (**121**) and other polyketides of the salicylaldehyde-type, such as heterocornols **130**–**132** and **135**–**137**, showed moderate activity. In the antimicrobial test, the MIC values ranged from 25 to 100 μg/mL in the pathogens *Staphylococcus aureus* and *Bacillus subtilis*. Interesting results were obtained in tests for lipid-lowering effects against oleic acid in HepG2 liver cells. Varioxiranol A (**39**), andytriol (**6**), and preshamixanthone (**11**) exhibited inhibitory effects at a dose of 10 μM and showed no toxicity up to 100 μM toward the tested cells. Moreover, preliminary tests of agropyrenol (**121**) and its derivatives **124**–**129** showed significant zootoxic and slight antimicrobial activities. This article also provides an overview of the laboratory syntheses of polyketides containing benzyl alcohol or salicylaldehyde functionalities (Table 3). We believe that a deeper understanding of the structural elements influencing biological properties offers a powerful tool for the controlled and rational design of new drugs or phytoherbicides based on natural polyketide derivatives.

## Figures and Tables

**Table 1:** Biological assays of agropyrenol (**121**) and its derivatives **124**–**129**.

Microorganism	Compound						
	121	124	125	126	127	128	129
*Cirsium arvense ^a^*	3	3	4	2	2	0	0
*Chenopodium albuma*	4	4	4	0	2	0	0
*Mercurialis annua ^a^*	4	2	4	0	0	0	0
*Setaria viridis ^a^*	0	0	1	0	0	0	0
*Sonchus oleraceus ^a^*	2	2	4	0	2	2	0
*Setaria viridis ^b^*	40.8 (0.3)	28.8 (1.1)	22.1 (1.7)	30.5 (2.2)	54.9 (0.1)	43.5 (0.9)	54.2 (0.4)
*Lycopersicon esculentum ^c^*	23.8 (11.9)	15.3 (7.1)	5.2 (2.7)	49.0 (21.0)	38.5 (14.0)	37.3 (9.9)	33.1 (9.7)
*Lemna minor ^d^*	6.4 (0.4)	5.3 (0.4)	2.5 (0.4)	7.3 (1.0)	7.1 (0.8)	8.5 (0.4)	8.8 (0.3)
*Lemna minor ^e^*	23.8 (2.8)	23.5 (1.8)	14.4 (4.1)	24.4 (3.5)	21.4 (2.3)	23.0 (1.6)	22.8 (1.9)
*Artemia salina ^f^*	0	0	88 (4)	0	0	23 (2)	0
*Geotrichum candidum*	-	-	+	-	-	-	-
*Bacillus subtilis*	-	-	+	-	-	-	-
*Escherichia coli*	-	-	-	-	-	-	-

*^a^* Data are expressed using a visual empiric scale from 0 = no symptoms to 4 = necrosis larger than 1 cm. *^b^* Data are expressed as a percentage of seed germination (SD in parentheses). *^c^* Data are expressed in mm (SD in parentheses). *^d^* Data are expressed in mg/L (SD in parentheses). *^e^* Data are expressed in mg/well (SD in parentheses). *^f^* Data are expressed as a percentage of dead larvae to the total (SD in parentheses).

**Table 2:** Summary of most active compounds for a range of bioactivities within this review.

Compound Source	Biological Activity	Cell Line or Microorganism	Biological Result	Ref.
Varitriol (**1**) *Emericella variecolor M75-2* (marine)	Cytotoxic activity	RXF 393 *renal cancer*	GI_50_ = 1.63 × 10^−7^ M	[11]
		*TD-47* *breast cancer*	GI_50_ = 2.10 × 10^−7^ M	
		*SNB* *CNS cancer*	GI_50_ = 2.44 × 10^−7^ M	
Varixanthone (**3**) *Emericella variecolor M75-2* (marine)	Antimicrobial activity	*Enterococcus faecalis*	MIC = 50.0 μg·mL^−1^	[11]
		*Bacillus subtilis*	MIC = 12.5 μg·mL^−1^	
		*Escherichia coli*	MIC = 12.5 μg·mL^−1^	
		*Proteus sp.*	MIC = 12.5 μg·mL^−1^	
Varioxiranediol A (**56**) *Emericella variecolor TJ29* (plant-derived)	Antibacterial activity	*MRSA*	MIC = 32 μg·mL^−1^	[43]
		*E. faecalis*	MIC = 32 μg·mL^−1^	
		*ESBL-E. coli*	MIC = 4 μg·mL^−1^	
		*P. aeruginosa*	MIC = 4 μg·mL^−1^	
		*K. pneumoniae*	MIC = 16 μg·mL^−1^	
Varioxiranediol B (**57**) *Emericella variecolor TJ29* (plant-derived)	Antibacterial activity	*E. faecalis*	MIC = 32 μg·mL^−1^	[43]
		*ESBL-E. coli*	MIC = 8 μg·mL^−1^	
		*P. aeruginosa*	MIC = 32 μg·mL^−1^	
Varioxirane (**2**) *Emericella variecolor TJ29* (plant-derived)	Antibacterial activity	*MRSA*	MIC = 64 μg·mL^−1^	[43]
		*ESBL-E. coli*	MIC = 32 μg·mL^−1^	
		*P. aeruginosa*	MIC = 32 μg·mL^−1^	
Heterocornol A (**130**) *Pestalotiopsis heterocornis* (marine)	Cytotoxic activity against human cancer cell lines	BGC–823 gastric carcinoma	IC_50_ = 32.9 μM	[68]
		H-460 lung carcinoma	IC_50_ = 37.3 μM	
		PC-3 prostate cancer	IC_50_ = 54.8 μM	
		SMMC-7721 hepatocellular carcinoma	IC_50_ = 26.4 μM	
Heterocornol B (**131**) *Pestalotiopsis heterocornis* (marine)	Cytotoxic activity against human cancer cell lines	BGC–823 gastric carcinoma	IC_50_ = 48.1 μM	[68]
		H-460 lung carcinoma	IC_50_ = 27.6 μM	
		PC-3 prostate cancer	IC_50_ = 31.5 μM	
		SMMC-7721 hepatocellular carcinoma	IC_50_ = 21.7 μM	
Heterocornol C (**132**) *Pestalotiopsis heterocornis* (marine)	Cytotoxic activity against human cancer cell lines	BGC–823 gastric carcinoma	IC_50_ = 28.2 μM	[68]
		H-460 large-cell lung carcinoma	IC_50_ = 18.7 μM	
		PC-3 prostate cancer	IC_50_ = 59.3 μM	
		SMMC-7721 human hepatocellular carcinoma	IC_50_ = 34.8 μM	
Compound **146** *Pestalotiopsis heterocornis* (marine)	Cytotoxic activity against human cancer cell lines	BGC–823 gastric carcinoma	IC_50_ = 80.2 μM	[68]
		H-460 lung carcinoma	IC_50_ = 87.3 μM	
		SMMC-7721 hepatocellular carcinoma	IC_50_ = 49.6 μM	
Agropyrenol (**121)** *Pestalotiopsis heterocornis* (marine)	Cytotoxic activity against human cancer cell lines	BGC–823 gastric carcinoma	IC_50_ = 77.4 μM	[68]
		H-460 lung carcinoma	IC_50_ = 24.5 μM	
		PC-3 prostate cancer	IC_50_ = 58.5 μM	
		SMMC-7721 hepatocellular carcinoma	IC_50_ = 52.0 μM	
Heterocornol F (**135**) *Pestalotiopsis heterocornis* (marine)	Cytotoxic activity against human cancer cell lines	BGC–823 gastric carcinoma	IC_50_ = 47.1 μM	[68]
		H-460 lung carcinoma	IC_50_ = 52.1 μM	
		PC-3 prostate cancer	IC_50_ = 70.6 μM	
		SMMC-7721 hepatocellular carcinoma	IC_50_ = 50.9 μM	
Heterocornol G (**136**) *Pestalotiopsis heterocornis* (marine)	Cytotoxic activity against human cancer cell lines	BGC–823 gastric carcinoma	IC_50_ = 15.0 μM	[68]
		H-460 lung carcinoma	IC_50_ = 53.3 μM	
		PC-3 prostate cancer	IC_50_ = 21.3 μM	
		SMMC-7721 hepatocellular carcinoma	IC_50_ = 20.2 μM	
Heterocornol H (**137**) *Pestalotiopsis heterocornis* (marine)	Cytotoxic activity against human cancer cell lines	BGC–823 gastric carcinoma	IC_50_ = 23.2 μM	[68]
		H-460 lung carcinoma	IC_50_ = 79.7 μM	
		PC-3 prostate cancer	IC_50_ = 83.5 μM	
		SMMC-7721 hepatocellular carcinoma	IC_50_ = 42.3 μM	
Vaccinol G (**148**) *Pestalotiopsis heterocornis* (marine)	Cytotoxic activity against human cancer cell lines	BGC–823 gastric carcinoma	IC_50_ = 51.2 μM	[68]
		H-460 lung carcinoma	IC_50_ = 57.9 μM	
		PC-3 prostate cancer	IC_50_ = 35.0 μM	
		SMMC-7721 hepatocellular carcinoma	IC_50_ = 44:4 μM	
Heterocornol M (**142**) *Pestalotiopsis heterocornis* (marine)	Cytotoxic activity against human cancer cell lines	BGC–823 gastric carcinoma	IC_50_ = 61.1 μM	[68]
		Hep G2 liver cancer	IC_50_ = 20.4 μM	
Heterocornol O/P (**144 / 145**) *Pestalotiopsis heterocornis* (marine)	Cytotoxic activity against human cancer cell lines	BGC–823 gastric carcinoma	IC_50_ = 35.0 μM	[68]
		Ichikawa	IC_50_ = 54.3 μM	
		Hep G2 liver cancer	IC_50_ = 42.0 μM	
		7860 kidney cancer	IC_50_ = 22.1 μM	
Compound **149** *Pestalotiopsis heterocornis* (marine)	Cytotoxic activity against human cancer cell lines	BGC–823 gastric carcinoma	IC_50_ = 82.1 μM	[68]
		Ichikawa	IC_50_ = 65.3 μM	
		Hep G2 liver cancer	IC_50_ = 94.2 μM	
Compound **150** *Pestalotiopsis heterocornis* (marine)	Cytotoxic activity against human cancer cell lines	BGC–823 gastric carcinoma	IC_50_ = 78.1 μM	[68]
		Ichikawa	IC_50_ = 58.5 μM	
		Hep G2 liver cancer	IC_50_ = 85.4 μM	

**Table 3:** Summary of synthesized metabolites within this review.

Compound	Ref.	Compound	Ref.
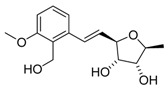 Varitriol ((+)-**1**)	[18,19,20,21,22,26,27,28,29] [25,33,34,35]	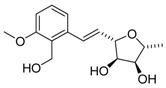 *ent*-Varitriol ((-)-**1**)	[15] [17] [32] [19]
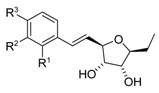 Analogues of varitriol **1a–h** **1a** R^1^=CH_2_OH, R^2^=OMe, R^3^=H **1b** R^1^=CH_2_OMe, R^2^=OMe, R^3^=H **1c** R^1^=OMe, R^2^=OEt, R^3^=H **1d** R^1^=OEt, R^2^=OEt, R^3^=H **1e** R^1^=OMe, R^2^=OMe, R^3^=OMe **1f** R^1^=Cl, R^2^=Cl, R^3^=H **1g** R^1^,R^2^=OCH_2_O, R^3^=H **1h** R^1^=OMe, R^2^=OMe, R^3^=H	[18]	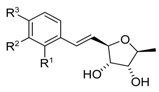 Analogues of varitriol **1i–s** **1i** R^1^=CH_2_OAc, R^2^=OMe, R^3^=H **1j** R^1^=H, R^2^=H, R^3^=H **1k** R^1^=OMe, R^2^=H, R^3^=H **1l** R^1^=H, R^2^=H, R^3^=OMe **1m** R^1^=OMe, R^2^=H, R^3^=OMe **1n** R^1^=CF_3_, R^2^=H, R^3^=H **1o** R^1^=H, R^2^= CF_3_, R^3^=H **1p** R^1^=H, R^2^=H, R^3^=Br **1r** R^1^=F, R^2^=H, R^3^=H **1s** R^1^=H, R^2^= H, R^3^=F	[30]
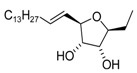 Analogue of varitriol **1t**	[18]	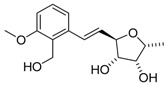 4-*epi*-Varitriol (4-*epi*-**1**)	[22]
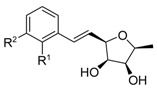 Analogues of 2,3-di*epi*-varitriol 2,3-*diepi*-**1a** R^1^=CH_2_OH, R^2^=OMe 2,3-*diepi*-**1b** R^1^=CH_2_OH, R^2^=H 2,3-*diepi*-**1c** R^1^=H, R^2^=OMe 2,3-*diepi*-**1d** R^1^=H, R^2^=H	[31]	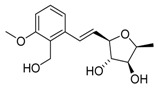 3-*epi*-Varitriol (3-*epi*-**1**)	[32]
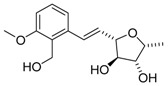 3-*epi*-*ent*-Varitriol (3-*epi*-*ent*-**1**)	[32]	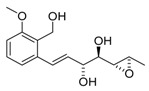 Varioxirane (**2**)	[33] [14]
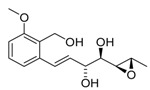 5,6-di*epi*-Varioxirane (5,6-di*epi*-**2**)	[14]	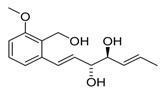 Andytriol (**6**)	[14]
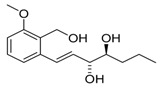 Varioxiranol A (**39**)	[41]	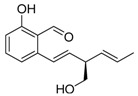 Pyricuol (**59**)	[54]
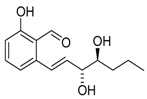 Pyriculol (**60**)	[52]	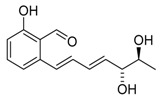 Pyriculariol (**64**)	[58]

## Data Availability

Not applicable.
